# Effects of Ginseng on Neurological Disorders

**DOI:** 10.3389/fncel.2020.00055

**Published:** 2020-03-20

**Authors:** Wei Hou, Yingping Wang, Peihe Zheng, Ranji Cui

**Affiliations:** ^1^Institute of Special Animal and Plant Sciences, Chinese Academy of Agricultural Sciences, Changchun, China; ^2^Jilin Provincial Key Laboratory on Molecular and Chemical Genetic, The Second Hospital of Jilin University, Changchun, China

**Keywords:** ginseng, neurological disorder, BDNF, depression, neuron

## Abstract

Ginseng (*Panax ginseng* Meyer), a famous traditional medicinal herb, has been widely used for many centuries. Numerous studies have shown that ginseng has a positive effect on the prevention and treatment of neurological disorders. In this review, we summarized the effects of ginseng in treating neurological diseases, particularly the anti-depressant effects of ginseng. Furthermore, its potential mechanism was also outlined. Therefore, this review may provide new insight into the treatment of ginseng on neurological diseases.

## Introduction

In recent years, medicinal plants have received extensive attention in the development of disease treatment. Ginseng (*Panax ginseng* Meyer, Araliaceous) is a famous traditional medicinal herb that has been used for many centuries. It is mainly distributed in Asian countries including China, Korea, and Japan (Shahrajabian et al., 2019). Ginseng is generally processed into fresh ginseng, red ginseng and white ginseng, according to the different technology (Majid, 2019). Numerous studies indicated ginseng promotes health and prevents potential disease (Rajabian et al., 2019), such as immune-modulatory, anti-inflammatory, lipid-lowering, anti-oxidation, anti-diabetic, anti-tumor, increase energy, restorative, anti-aging, anti-depression, inhibit or delay the neurodegenerative process, improving memory and perceptual systems (Ong et al., 2015). Due to lots of pharmacological effects, ginseng has attracted much attention world widely.

As our global population ages, hundreds of thousands of older adults suffer neurological disorders disease. Most medication has side effects for central nervous system diseases (Dording and Boyden, 2019). However, ginseng as a medicinal plant has fewer side effects in treating diseases, and it has certain advantages in treating central nervous system disease (Shahrajabian et al., 2019). Scholars had reported that red ginseng inhibited neuronal damage and ginseng inhibited or delayed Parkinson's disease (PD), Huntington's disease (HD), and Alzheimer's disease (AD) (Cho, 2012; Iqbal et al., 2019). In addition, the antidepressant activity of ginseng and its ginsenosides has been widely reported. In order to better study and apply ginseng in central nervous system diseases, we describe the neuropharmacology of ginseng in this paper (Table 1).

**Table 1:** Effects and mechanisms of active ingredients of ginseng on the central nervous system disease.

**Effects**	**Cells/Animals**	**Dose and route**	**Model**	**Active ingredients**	**Mechanism**	**References**
Anti-depression	Adult male C57BL/6J mice (8–10 weeks old) and male CD1 mice (50 weeks old)	5, 10, 20, and 40 mg/kg (intraperitoneally)	Chronic social defeat stress	Rg5	Activating hippocampal BDNF system	Xu et al., 2017
Anti-depression	Adult male C57BL/6J mice (8–10 weeks old) and male CD1 mice (50 weeks old)	10 and 20 mg/kg (intraperitoneally)	Chronic social defeat stress	Rg3	Activating hippocampal BDNF system	You et al., 2017
Anti-depression	Weighing 20–25 g (National Laboratory Animal Center, Mahidol University)	10, 20, 40, and 80 mg/kg G115 (oral gavage)	Ethanol-treated	G115	Increasing BDNF levels in the hippocampus and prefrontal cortex	Boonlert et al., 2017
Anti-depression	Adult male C57BL/6J mice (8–10 weeks old)	2.5, 5, 10, and 20 mg·kg^−1^ (intraperitoneally)	Chronic mild stress	Rg1	Activating BDNF signaling pathway and up-regulation of hippocampal neurogenesis BDNF-TrkB	Jiang et al., 2012
Anti-depression	Male ICR mice (18–22 g)	0.25 and 1 mg/kg (oral gavage)	Lipopolysaccharide-induced depression	Ginseng sesquiterpenoids	Modulating BDNF/TrkB and Sirt1/NF-κB signaling pathways.	Wang et al., 2018
Anti-depression	ICR albino female mice	Rb1 2.5, 5, and 10 mg/kg/day; compound K 1.25, 2.5, 5 mg/kg/day (intraperitoneally)	Ovariectomy	Rb1, compound K	Modulating 5-HT(2A) receptors	Yamada et al., 2011
Anti-depression	Female Sprague Dawley rats aged 10 weeks (body weight: 180–200 g)	200 and 400 mg/kg per os	Ovariectomized and restraint stressed	White ginseng powder	Increasing hippocampal 5-HT level	Jang et al., 2019
	Male ICR mice (22–24 g) and Wistar rats (200–220)	4, 8, and 10 mg/kg per os	Chronic unpredicted mild stress	ginsenoside Rb1	Modulating serotonergic, noradrenergic and dopaminergic systems.	Wang et al., 2017
Anti-depression	Male C57BL/6 J mice weighing 20–25 g	20 mg/kg/d (intragastric gavage)	Sucrose Preference Test Tail Suspension Tests Forced Swim Tests	Ginseng fruit saponin	Regulating 5-HT concentrations	Liu L. et al., 2019
Anti-depression	Adult Kunming mice (male) and Sprague-Dawley rats (male)	5, 10, 20, and 40 mg·kg^−1^ (intragastric gavage)	Chronic-unpredictable-mild-stress Gonadectomized model	Rg1	Modulating HPA and the HPG axis	Mou et al., 2017
Anti-depression	BALB/c male mice 20–22 g	20, 40, and 80 mg/kg (intragastric gavage)	Chronic unpredictable mild stress	dammarane sapogenins	Regulating neurotransmitters and hypothalamic–pituitary–adrenal axis	Jiang et al., 2018
Anti-depression	Adult male C57BL/6 mice 8–10 week of age	75, 150, and 300 mg/kg (intragastric gavage)	Chronic restraint stress	*Panax ginseng* aqueous extract	Inhibiting hypothalamo-pituitary-adrenal axis	Choi et al., 2018
Anti-depression	Human neuroblastoma SHSY-5Y cells (passages 20–30)	Rb1 100 ng/ml Rg3 100 ng/ml		Rb1 and Rg3	Glucocorticoid	Kim et al., 2010
Neuroprotection	The wildtype and the Nrf2^−/−^ mice had a C57BL/6 genetic background (10–18 weeks old)	100 mg/kg/day(gavage)	Permanent distal middle cerebral artery occlusion	The standardized Korean red ginseng, a water-soluble extract	Nrf2-dependent	Liu L. et al., 2019
Neuroprotection	Young (4 months), middle-aged (12 months) and aged mice (24 months) with a C57BL/6J background	0.5, 1, 5, and 10 mg/kg (gavage)	Middle cerebral artery occlusion	Rb1	Anti-Oxidant Signaling	Dong et al., 2017
Neuroprotection	Male ICR mice, weighing 25–30 g	5, 20, or 40 mg/kg (intraperitoneal injection)	Middle cerebral artery occlusion	ginsenoside Rb1	Suppressing neuroinflammation induction of MMP-9 and NOX4-derived free radicals	Chen et al., 2015
Neuroprotection	Male Sprague–Dawley rats weigh 270–320 g	30 mg/kg (intraperitoneally)	Middle cerebral artery occlusion	Rd	Up-regulating GLT-1 expression through PI3K/AKT and ERK1/2	Zhang et al., 2013
Neuroprotection	PC12 cells	0.1–100 μM		Ginsenoside Rd	Promoting the neurite outgrowth via ERK and AKT dependent signaling pathways	Wu et al., 2016
Neuroprotection	Male SD rats PC12 cells	1, 2.5, and 5 mg·kg^(−1)^·d^(−1)^ (intraperitoneally) 25, 50, and 100 μmol/L	Transient middle cerebral artery occlusion Oxygen glucose deprivation	ginsenoside Rd	Activating the PI3K/Akt and ERK1/2	Liu X. Y. et al., 2015
Neuroprotection	Male BALB/c mice (25–30 g) Mice astrocytes	20 and 40 mg/kg (intraperitoneally) 0, 2.5, 5, 10, and 20 μM	Transient middle cerebral artery occlusion H_2_O_2_-induced apoptosis	Rg1	Preventing the astrocytes from apoptosis.	Sun et al., 2014
Neuroprotection	Male Wistar rats aged 45–60 d old and body weights from 250 to 300 g	25 mg^(−1)^·d^(1)^ (intraperitoneally)	Middle cerebral artery occlusion	Ginseng total saponins	Protecting brain cell	Zheng et al., 2011
Neuroprotection	Immortalized murine BV2 microglial cells, C57BL/6 mice 10–11 weeks old	Compound K (25, 50, and 75 μM), 30 mg/kg (intraperitoneally)	Systemic inflammation Middle Cerebral Artery Occlusion	Compound K	Inhibiting activation of microglial	Park et al., 2012
Neuroprotection	Male Sprague-Dawley rats weighing between 250 and 300 g	20 mg/kg	Subarachnoid hemorrhage-induced brain injury	Rb1	Reducing arterial vasospasm and brain edema	Li et al., 2011
Improve cognition	Male C57BL/6 mice (10 weeks, 25–27 g)	50 and 100 mg/kg (oral gavage)	Scopolamine-induced memory deficits	ginsenoside Rg3-enriched ginseng ethanol extract	Inhibiting of acetylcholinesterase activity and NF-κB signaling	Kim J. et al., 2016
Improve cognition	Male ICR mice (28–30 g)	5, 10, and 20 mg/kg (oral gavage)	Scopolamine-induced memory deficits	Rh3 and Rg5	Inhibiting AChE activity and increasing BDNF expression and CREB activation	Kim et al., 2013
Improve cognition	Male ICR mice, 6 months of age	5 and 10 mg/kg/day(oral gavage)		Rh1	Enhancing cell survival and expression of BDNF	Hou et al., 2014
Improve cognition	Male C57BL/6J mice (12-month-old)	0.1, 1, and 10 mg/kg (intraperitoneally)		Rg1	Regulating the PI3K/AKT pathway, altering apical spines and facilitating hippocampal LTP	Zhu et al., 2015
Anti-AD	The mouse hippocampal neuronal HT22 cell line The littermates obtained through the crossing of male 5XFAD mice and female B6SJL/F1 mice	1, 10, and 100 μg/mL 100 mg/kg (oral gavage)	Amyloid beta-mediated mitochondrial dysfunction	red ginseng MeOH extract	Mitochondria-related	Shin et al., 2019
Anti-AD	PC12 cells	β-sitosterol, and stigmasterol 0.1 and 1.10 μM Linoleic acid 10, 50, and 100 μM	Aβ_25−35_ treatment	linoleic acid, β-sitosterol, and stigmasterol	Regulating oxidative stress, apoptotic responses, and pro-inflammatory mediators	Lee et al., 2018b
Anti-AD	Male Wistar rats 400 ± 50 g, human neuroblastoma SH-SY5Y cells	40, 80, and 160 mg/kg·d^−1^ intraperitoneally 0.03, 0.1, 0.3, or 1 μg/mL	Aβ_25−35_ treatment	PGL-1	Reducting NO concentration and NOS activity	Luo et al., 2018
Anti-AD	Sprague-Dawley (SD) embryo rat cortical neurons	5, 10, 20, and 40 μmol	Aβ_25−35_ treatment	Rb1	Attenuating expression of JNK/p38 MAPK	Song et al., 2008
Anti-AD	APP transgenic mice, at the age of 10 months	10 mg/kg (intraperitoneally)		Rd	Inhibiting the transcription activity of NFκB	Liu J. et al., 2015
Anti-AD	Neuroglial cell line NG108-15	2, 4, 8, 16, and 32 μg/mL	Aβ_25−35_ treatment	Rg1	Suppressing the signaling transduction pathway of TLR3 and TLR4, inflammation factors	Zhao et al., 2014
Anti-AD	Hippocampal neurons from the Sprague-Dawley neonates	50 μM	Aβ_25−35_ treatment	Rg1	Activating Akt and ERK1/2 signaling	Huang et al., 2016
Anti-AD	Male Wistar rats weighing 180–220 g (aged 7 weeks)	2 g /kg/day (oral gavage)	d-galactose and AlCl3 treatment	total ginsenosides water extracted	Restoring the dysfunction of various neurotransmitters	Zhang Y. et al., 2016
Anti-AD	Adult male Sprague-Dawley rats (280–300 g)	1, 0.5, and 0.25g/kg (oral gavage)	Advanced glycation end product treatment	Ginseng water extracts	RAGE/NF-κB	Tan et al., 2015
Anti-PD	The dopaminergic cell of OF1/SPF embryos mice	Rd 1–10 μM Re 1–10 μM	CCl4 treatment	Rd and Re	Inhibiting oxidative stress and inflammation	Zhang X. et al., 2016
Anti-PD	SH-SY5Y cells Adult male C57BL/6J mice (22–25 g)	1 and 10 μM 10 mg/kg (intraperitoneally)	1-methyl-4-phenylpyridinium treatment 1-methyl-4-phenyl-1,2,3,6-tetrahydropyridine treatment	Ginsenoside Rd	Antioxidant effects and mitochondrial function preservation	Liu Y. et al., 2015
Anti-PD	C57BL/6J mice (6–8 weeks old, male, weighing 16–25 g) Rat pheochromocytoma PC12 cells	5, 10, and 20 mg/kg (intraperitoneally) 20 μM	1-methyl-4-phenyl-1,2,3,6-tetrahydropyridine treatment 1-methyl-4-phenylpyridinium treatment	Rg1	Wnt/β-catenin	Zhou et al., 2016
Anti-PD	Adult female Wistar rats (250–300 g)	10 mg/kg, 10 mg/ml (intraperitoneally)	Lipopolysaccharide treatment	Rg1	Glucocorticoid receptor signaling pathway	Sun et al., 2016
Anti-PD	Male C57BL/6 mice (9 weeks old)	100 mg/kg (oral gavage)	1-methyl-4-phenyl-1,2,3,6-tetrahydropyridine treatment	Korean Red Ginseng water extract	Alleviates protein expression profiles	Kim D. et al., 2016
Anti-PD	Male Wistar rats aged 3 months and weighing 240–280 g	20 mg/kg (intraperitoneally)	Lipopolysaccharide treatment	Rb1	Inhibiting inflammation protecting dopaminergic neuron	Li et al., 2019
Anti-HD	Male Sprague-Dawley rats weighing 240 ± 10 g	5, 10, and 20 mg/kg (oral gavage)	3-nitropropionic acid treatment	Protopanaxtriol	Anti-oxidant	Gao et al., 2015
Anti-HD	Medium spiny striatal neuronal cultures from the YAC128 HD mouse model	Rb1 0.01 and 0.1 μM; Rc 0.01 μM; Rg5 1.0 μM	Glutamate stimulation	Rg5, Rb1 and Rc	Anti-apoptosis	Wu et al., 2009

*MAPK, mitogen-activated protein kinase; ERK, extracellular signal-regulated kinase; TLR, toll-like receptor; RAGEs, the receptors for advanced glycation end products; NF-κB, nuclear factor-kappa-light-chain-enhancer of activated B cell; JNK, c-Jun N-terminal kinase; BDNF, brain-derived neurotrophic factor; TrkB, brain-derived neurotrophic factor; 5-HT, 5-hydroxytryptamine; HPA, hypothalamic–pituitary–adrenal axis; HPG, hypothalamic pituitary gonadal axis; Nrf2, NF-E2-related factor 2; MMP-9, matrix metalloproteinase-9; NOX, nicotinamide adenine dinucleotide phosphate oxidase; GLT-1, glutamate transporter 1; PI3K/AKT, (phosphatidylinositol-3-kinases)/(protein-serine-threonine kinase); acetylcholinesterase; LTP, long-term potentiation; AChE, acetylcholinesterase; CREB, cAMP-response element-binding protein; AD, Alzheimer's disease; HD, Huntington's disease; PD, Parkinson's disease*.

## Active Constituents

Various constituents of ginseng possess pharmacological activity including ginsenosides, ginseng polysaccharides, ginseng polypeptides, volatile oils, polyacetylenes, organic acids, and esters (Jin et al., 2019). In all active constituents, ginsenosides are the most pharmacologically active constituents and they are the main focus of ginseng research. Usually, ginsenosides are grouped into two major groups based on their chemical structures, protopanaxadiol (PPD) and protopanaxatriol (PPT). The PPD group includes Rb1, Rb2, Rc, Rd, Rg3, Rh2, and Rh3; The PPT group includes Re, Rf, Rg1, Rg2, and Rh1 (Rajabian et al., 2019).

## The Treatment of Ginseng in Neurological Disorders

### The Role of Ginseng on Depression

Depression is characterized by sleep disturbance, loss of interest, lack of energy, anxiety, and suicidal thoughts (Zhang et al., 2019). Treatments for depression include electroshock therapy, drug therapy, and psychotherapy. However, for major depression disorder, medication is essential. Existing antidepressants like these NA reuptake inhibitors (NRIs) and selective serotonin reuptake inhibitors (SSRIs) possess latency period of antidepressant efficacy (Farber and Goldberg, 2004; Aleksandrova et al., 2019) and three major classical antidepressants possess side effects (Gillman, 2007; Wang et al., 2017). Moreover, currently available ADs are not effective enough (Haenisch and Bonisch, 2011) and mental health resources are not sufficient (Smith, 2014). Therefore, it is necessary to find antidepressants with quicker effects and fewer side effects. The history of ginseng used to treat central nervous system diseases can be traced back to the Eastern Han Dynasty (Wang et al., 2017). In addition, many components of ginseng exert antidepressant effects by acting on different targets.

**Figure 1:**
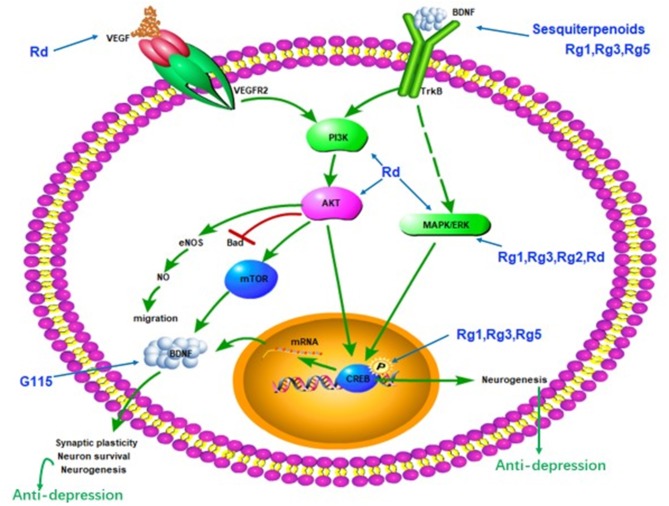
Schematic diagram summarizing the antidepressant effect of ginseng via the BDNF-TrkB signal path. G115, ginseng extract G115; BDNF, brain-derived neurotrophic factor; VEGF, vascular endothelial growth factor; PI3K, phosphoinositide 3-kinase; AKT, protein kinase B; mTOR, mammalian target of rapamycin; MAPK, mitogen-activated protein kinase; ERK, extracellular regulated protein kinases; CREB, cAMP-response element-binding protein.

Impairment of synaptic plasticity was one of the pathogenesis of depression. Synaptic regulators, such as BDNF is important in the treatment of depression (Castren and Rantamaki, 2010). The hippocampus and prefrontal cortex are brain regions associated with depression. Stress and depression reduce the expression and function of BDNF in these two sites. The level of BDNF in the blood of depressed patients is also reduced (Krishnan and Nestler, 2008; Bocchio-Chiavetto et al., 2010). Antidepressants can increase the expression of BDNF (Molteni et al., 2006; Reus et al., 2013; Duman et al., 2016). Ginsenoside Rg5 restored the chronic social defeat stress (CSDS)-induced decrease in hippocampal BDNF signaling cascade (Xu et al., 2017). Ginsenoside Rg3 improved depression-like behavior in the depression model test. Rg3 also reverse the CSDS-induced decrease of BDNF signaling pathway in the brain (You et al., 2017). A possible cause of ethanol-induced depression is a reduction in BDNF levels in the brain. G115 significantly increased BDNF levels in the hippocampus and prefrontal cortex of ethanol-induced depression mice (Boonlert et al., 2017). The antidepressant effect of Rg1 and Ginseng sesquiterpenoids may be related to the enhancement of the BDNF-TrkB signaling pathway (Jiang et al., 2012; Wang et al., 2018). Rg1 increases the hippocampal BDNF level and phosphorylation of downstream molecules ERK and CREB in the chronic mild stress mouse model. In addition, the antidepressant effect of Rg1 can be blocked by the BDNF receptor trkB inhibitor K252a (Jiang et al., 2012). These results suggest that the active ingredient of ginseng may exert antidepressant effects through enhanced BDNF-TrkB signaling pathways (Figure 1).

Serotonin or 5-hydroxytryptamine (5-HT), known as Monoamine neurotransmitters. The decrease in blood serotonin levels is closely related to the occurrence of depression (Sekiyama et al., 2013; Zahn et al., 2015). Ginsenoside Rb1 and compound K showed an antidepressant-like effect after the ovariectomy during the forced swimming test. 5-HT_2A_ receptors mediated the antidepressant-like effect of Rb1 (Yamada et al., 2011). White ginseng and Ginseng fruit saponin increased 5-HT concentration and improved the depression-like behavior of mice (Jang et al., 2019; Liu M. et al., 2019). High doses of 5-HTP, a precursor of serotonin, can cause head-twitches in mice, and antidepressants that increase serotonin can aggravate this symptom. Similarly, Ginsenoside Rb1 can significantly increase the number of head-twitches caused by 5-HTP and Tryptophan hydroxylase inhibitors prevent the antidepressant effects of ginsenosides Rb1. So, ginsenoside Rb1 may have an effect of increasing 5-HT (Wang et al., 2017). Most blood serotonin is supplied by gastrointestinal enterochromaffin (EC) cells and stored in EC cells (McLean et al., 2007; Bertrand and Bertrand, 2010). Intestine-derived serotonin acts in the brain through the gut-brain axis and plays an important role in diseases associated with serotonin, including depression (Saldanha et al., 2009; Albert et al., 2012). Gintonin (intragastric administration) stimulates the release of serotonin from mouse EC cells via lysophosphatidic acid (LPA) receptor-mediated [Ca^2+^]i transients, followed by elevated plasma serotonin levels, reducing depression-like behavior (Kim et al., 2017). Compared with ginsenosides, dammarane sapogenins (DS), the hydrolysate of ginsenosides, is more easily absorbed by the body and possess stronger biological activity. DS showed significant antidepressant effects in different depression models and significantly increased hippocampal serotonin levels (Jiang et al., 2018). In conclusion, the increase in serotonin levels may be another mechanism by which ginseng active ingredients exert antidepressant effects.

**Figure 2:**
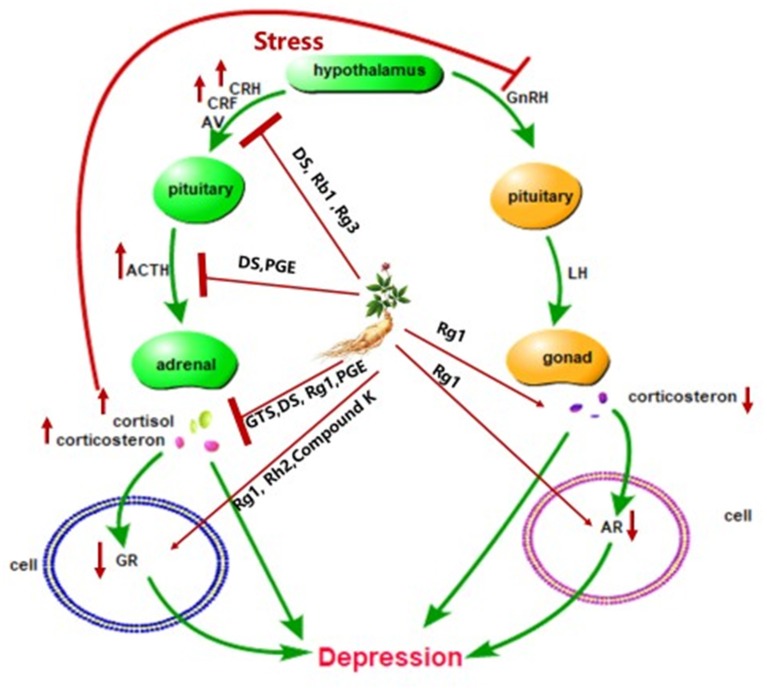
Schematic diagram summarizing the antidepressant effect of ginseng via the HPA axis. CRH, corticotrophin-releasing hormone; AV, arginine vasopressin; ACTH, adrenocorticotropic hormone; CRF, corticotropin-releasing factor; GR, glucocorticoid receptor; AR, androgen receptor; GnRH, gonadotropin-releasing hormone; LH, luteinizing hormone; DS, dammarane sapogenins; PGE, *Panax ginseng* extract; GTS, ginseng total saponins.

The hyperactivity of the hypothalamic-pituitary-adrenal (HPA) axis caused by stress is related to the pathogenesis of depression (Kawabata et al., 2010), accompanied by the dysfunction of hypothalamic-pituitary-gonadal (HPG) axis (Mou et al., 2017). HPG axis was weakened in depressed individuals (Jin et al., 2019) and testosterone can improve depression mood in clinical therapy (Kanayama et al., 2007). HPA axis is the biological system responsible for stress response. When stress occurs, the hypothalamic releases the adrenocorticotropic releasing hormone and arginine vasopressin to the pituitary gland to produce the adrenocorticotropic hormone, which stimulates the adrenal gland to produce cortisol or corticosterone (Lee and Rhee, 2017; Shapero et al., 2019; Figure 2). Cortisol plays an important role in learning, memory, and emotion (Sapolsky et al., 2000). However, excessive cortisol produced by stress can alter the function of the brain associated with depression, like the hippocampus, prefrontal cortex, and the amygdala (McEwen, 2008; Shapero et al., 2019). Rg1 alleviated anhedonia, hopelessness and improved sleep disruption through the modulation levels of corticosterone, testosterone, androgen receptor (AR), and glucocorticoid receptor (GR) in the chronic-unpredictable-mild-stress model and the gonadectomized model (Mou et al., 2017). *Panax ginseng* extract exerted an antidepressant-like effect by inhibiting the HPA axis in depressed mice (Choi et al., 2018). Treatment with DS reverses the rise in corticotropin-releasing factor, adrenocorticotropic hormone, and cortisol in the serum of mice (Jiang et al., 2018). The excessive corticotropin-releasing factor is associated with depression and anxiety (Arborelius et al., 1999). In addition, dexamethasone-induced cytotoxicity was blocked by Rb1 and Rg3 (Kim et al., 2010). Increased glucocorticoid levels as a result of stress can reduce the expression of glucocorticoid receptors (GR), which then block GR–TrkB interaction and BDNF (Chiba et al., 2012). Rh2 and Compound K, as a ligand for GR, can activate GR (Lee and Ji, 2014). Therefore, the active ingredients of ginseng may exert antidepressant effects through the HPA axis.

Inflammatory cytokine disorders may lead to depression. Our previous study elaborated the anti-depression mechanism of ginseng through the immune system and ginsenosides Rg1 exert antidepressant effects through multiple signaling pathways of cells (Jin et al., 2019). It is worth mentioning that ginsenosides Rg1 seems to exert antidepressant effects in a variety of ways (Figures 1, 2). Such as the BDNF-TrkB signaling pathway, HPA axis and immune system. Moreover, Rg1 is the main component of ginseng with a variety of biological activities, such as promoting neurogenesis and neurotrophin expression, acting on glucocorticoid receptors, and sex hormone receptors (Mou et al., 2017). Therefore, Rg1 may become a new generation of antidepressants.

### The Role of Ginseng on Neuronal Damage

Because of its high oxidative metabolic rate and low antioxidant capacity, the brain is highly susceptible to oxidative stress, leading to neuroinflammation, and damage. Heat stress can trigger oxidative stress and inflammatory factor release, leading to neurogenesis damage, and neuronal death. Red Ginseng (RG) mitigated the release of proinflammatory mediators and neuronal damage induced by heat stress in the rat (Iqbal et al., 2019). Trimethyltin (TMT) was a strong neurotoxin that can induce hippocampal neuronal injury. Rh2 and Rg3 decreased TMT-induced neurotoxicity and oxidative stress by enhancing PI3K/Akt signaling pathways and inhibiting ERK activation (Hou et al., 2018). Ginsenoside Rg1 protects against H_2_O_2_-induced neuronal damage. H_2_O_2_ treatment increased reactive oxygen species (ROS) production and induced hippocampal neuron damage. NADPH oxidase 2 (NOX2) mainly produced ROS in the brain. Rg1 may reduce NOX2-mediated ROS generation and inhibit neuronal damage (Xu et al., 2019). Rb2 possesses neuroprotective effects by suppressing glutamate-induced neurotoxicity. Glutamate was the prime excitatory neurotransmitter. Excessive glutamate concentration in the brain can cause neurotoxicity (Kim et al., 2019). The degree of the inflammatory response is positively correlated with the degree of brain damage. Rb1 increased *Streptococcus pneumoniae* clearance and cell survival by increasing the expression of BDNF and anti-apoptotic factors (Lee et al., 2018a). In a word, Ginseng may protect neuron from neurotoxicity and other damage

Stroke was a brain rupture or obstruction in the brain's blood supply. It was divided into two types: ischemic stroke and hemorrhagic stroke. Cerebral ischemia is a devastating disease with high mortality and disability primarily due to infarction. Korean Red Ginseng attenuated long-term brain damage and protected neuro in a permanent cerebral ischemia model. The transcriptional factor Nrf2 played an important role in the long-term recovery of permanent cerebral ischemic damage and the neuroprotection of ginseng (Liu L. et al., 2019). The long-term pretreatment with ginsenoside reduced brain damage caused by middle cerebral artery occlusion (MCAO). Ginsenoside Rb1 was beneficial for treating cerebral ischemia (Dong et al., 2017) and Rb1 protected blood-brain barrier (BBB) from ischemic stroke (Chen et al., 2015). Ginsenoside Rd protected against ischemic cerebral damage both *in vitro* and *in vivo*. Rd reduced the level of glutamate by increasing GLT-1 expression via ERK1/2 and PI3K/AKT pathways (Zhang et al., 2013). GAP-43, a neuron-specific protein, is related to regeneration and axonal outgrowth, Moreover, Rd could promote neurite outgrowth by upregulating GAP-43 expression mediated by PI3K/AKT and MAPK/ERK pathways, which is beneficial for treating ischemic stroke (Wu et al., 2016). Besides, Rd not only can activate PI3K/Akt and ERK1/2 signaling pathways but also can increase the expression of VEGF and BDNF in PC12 cells subjected OGD/reperfusion (Liu X. Y. et al., 2015). Ginsenoside Rg1 significantly reduced brain edema and infarct volume after MCAO by preventing of astrocytes from apoptosis (Sun et al., 2014). Ginseng total saponins (GTS) can protect brain cells, induce endogenous Neural stem cells to proliferate, and improve neurological function deficits after ischemic injury (Zheng et al., 2011). Compound K, a ginseng saponin metabolite, reduced the ischemic cerebral volume caused by MCAO and inhibited activation of microglia in the ischemic cortex (Park et al., 2012). In addition, Ginsenoside Rb1 reduced arterial vasospasm and brain edema and improved neurobehavioral function in the brain-injured rat (Li et al., 2011). All of these suggest that ginseng can protect the brain against ischemic stroke.

### The Role of Ginseng on Neurodegenerative Disorders

Neurodegenerative disorders are multifactorial debilitating diseases and gradual loss of neuronal structure and function, like cognitive impairments, Alzheimer's disease (AD), Huntington's disease (HD), and Parkinson's disease (PD). Age and genetic mutations can also cause neurodegenerative diseases (Huang et al., 2019). Many pieces of research showed the active ingredients of ginseng improved neurodegenerative diseases by different pathways.

The decline of central cholinergic nerve function caused cognitive disorder. Ginsenoside Rg1 and Rb1 reversed the decline by increased the expression of acetylcholine(Ach), improving learning and memory (Lai et al., 2018). Ginsenoside Rg3-enriched ginseng extract alleviated scopolamine-induced memory damage through the inhibition of the NF-κB pathway and acetylcholinesterase (AchE) activity (Kim J. et al., 2016). Ginsenoside Rh3 and Rg5 may improve memory deficit induced by scopolamine in mice. They inhibited AChE activity and increased BDNF expression (Kim et al., 2013). Furthermore, Long-term administration of ginsenoside Rg1 and Rh1 can enhance learning ability by increasing BDNF (Hou et al., 2014; Zhu et al., 2015).

**Figure 3:**
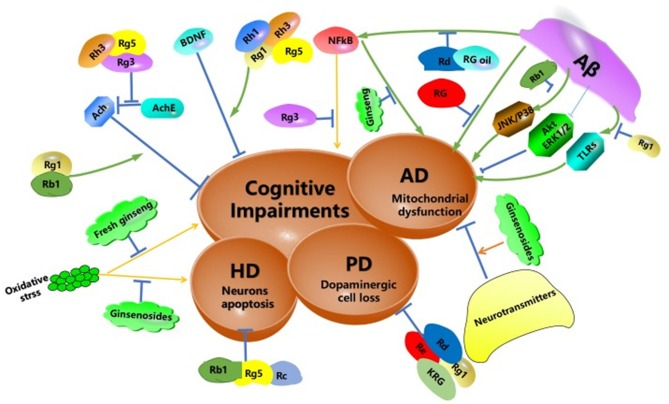
Schematic diagram summarizing the potential protective effects of ginseng and its constituents against neurodegeneration. Aβ, amyloid beta peptide; TLRs, toll-like receptors; AD, Alzheimer's disease; HD, Huntington's disease; PD, Parkinson's disease; NF-κB, nuclear factor-kappa-light-chain-enhancer of an activated B cell; JNK, c-Jun N-terminal kinase, AChE, acetylcholinesterase; ERK, extracellular signal-regulated kinase; RG, red ginseng.

The deposition of the amyloid beta-peptide (Aβ) is associated with AD. However, the mitochondrial deficiency was also considered a mediator or trigger for AD development. RG can reduce Aβ-induced mitochondrial dysfunction both *in vitro* and *in vivo*. The results showed RG may be a mitochondria-targeting agent for the treatment of AD (Shin et al., 2019). In addition, red ginseng oil decreased amyloid-beta peptide fragment 25–35 (Aβ_25−35_)-induced neuronal inflammation and apoptosis by MAPK/NF-κB pathway (Lee et al., 2018b). A glycoprotein was extracted from ginseng, which named PGL-1. PGL-1 could alleviate the memory impairment of rats induced by Aβ_25−35_. The glycoproteins might be a promising anti-AD reagent (Luo et al., 2018). Ginsenoside Rb1 markedly decreased tau protein hyperphosphorylation through JNK/p38 MAPK pathway (Song et al., 2008). Ginsenoside Rd, as an alternative treatment for AD patients, could improve cognize ability in amyloid β-protein precursor (APP) transgenic(Tg) mice by inhibiting NF-κB (Liu J. et al., 2015). Ginsenoside Rg1 has anti-neurodegenerative effects on Aβ_25−35_ induced hippocampus and neurite damage *in vitro*. Activating Akt and ERK1/2 signaling may mediate enhanced neurite outgrowth and reduced Aβ_25−35_ induced damage by Rg1 (Huang et al., 2016). In addition, toll-like receptors (TLRs), were an essential defense system for the mammalian host. In AD patients, cells express more TLRs than normal in the brain. Rg1 suppressed the signaling transduction pathway of TLR3 and TLR4 and decreased the inflammation factors (Zhao et al., 2014). Ginsenosides Rg1 can inhibit the signal transduction pathway of TLRs. Cognitive-related neurotransmitters disorders can be restored by ginsenosides (Zhang Y. et al., 2016). Moreover, ginseng extracts inhibited memory impairment of AD rats. The neuroprotective effects of Ginseng extracts may be related to the RAGE/NF-κB signal path (Tan et al., 2015). These findings suggest that ginseng can act on a variety of signaling pathways to improve the symptoms of Alzheimer's disease (Figure 3).

Degeneration of dopaminergic neurons was the main characteristic of PD. High levels of Carbon tetrachloride (CCl_4_) affected the nervous system, including nigrostriatal dopaminergic nerve cells. Interestingly, ginsenosides Rd and Re reduced cell loss and degeneration (Zhang X. et al., 2016). In addition, Rd significantly attenuates SH-SY5Y cells injury in a PD model *in vitro*. The mechanism may be due to its mitochondrial function preservation and antioxidant effects (Liu Y. et al., 2015). Ginsenoside Rg1 decreased dopaminergic cell loss via Wnt/β-catenin pathway (Zhou et al., 2016). And Rg1 inhibited inflammation-induced dopaminergic neuronal degeneration by glucocorticoid receptor signaling pathway (Sun et al., 2016). Korean Red Ginseng (KRG) also suppressed MPTP-induced behavioral dysfunction and dopaminergic neuronal death (Kim D. et al., 2016). Neuroinflammation caused by the microglial overactivation is associated with PD. Ginsenoside Rb1 inhibited lipopolysaccharide-induced microglial overactivation and protected dopaminergic neurons (Liu L. et al., 2019). Ginseng extract showed a partial therapeutic effect on the rat model of PD induced by the intrastriatal injection of rotenone (Khadrawy et al., 2016).

HD was a hereditary neurological disease caused by cytosine-adenine-guanine triplet expansion. HD patients exhibited abnormal body movements, personality disorders and cognitive impairments (Huang et al., 2019). Ginseng extract protopanaxatriol protects rat from oxidative stress induced by 3-nitropropionic (a model agent used to mimic the characteristics of HD) (Gao et al., 2015). Ginsenosides Rg5, Rb1, and Rc block glutamate-induced apoptosis of YAC128 medium spiny neurons (Wu et al., 2009). The potential protective effects of ginseng and its constituents against neurodegeneration were outlined in Figure 3.

## Conclusion

Ginseng can be used as medicine and also be used as food. Due to the few side effects, consumers and researchers paid more attention to it (Kitts and Hu, 2000). Over the years, ginseng had been increasingly studied in neurological diseases. Ginseng and its saponins were effective drugs for the treatment of brain diseases, like depression, neuronal damage, AD, PD, and HD. Ginseng can act on neurotransmitters (serotonin, acetylcholine), hormones (cortisol, corticosterone, testosterone) and receptor (androgen receptor, glucocorticoid receptor), brain-derived neurotrophic factors, and a variety of intracellular signaling molecules. It is worth mentioning that Ginsenoside Rg1, as the main component of ginseng, possess plenteous biological activity, like anti-depression, anti-Alzheimer's disease, anti-Parkinson's disease and protect neurons. The antidepressant effect of Rg1 is the same as imipramine. So, Rg1 has great development potential. In summary, ginseng has a positive effect on the treatment of brain diseases and it deserves further research and development.

## Author Contributions

WH conceived the idea, wrote the manuscript, and edited the manuscript. YW and PZ participated in the discussion of the manuscript. RC revised the manuscript.

### Conflict of Interest

The authors declare that the research was conducted in the absence of any commercial or financial relationships that could be construed as a potential conflict of interest.

## Abbreviations

BDNF: brain-derived neurotrophic factor
CSDS: chronic social defeat stress
TrkB: Tyrosine Kinase receptor B
ERK: extracellular regulated protein kinases
CREB: cAMP-response element-binding protein
PI3K/Akt: (phosphatidylinositol-3-kinases)/(protein-serine-threonine kinase)
5-HT: 5-hydroxytryptamine
EC: enterochromaffin
DS: dammarane
5-HTP: 5-hydroxytryptophan
HPA: hypothalamic–pituitary–adrenal axis
GR: glucocorticoid receptors
TMT: trimethyltin sapogenins
ROS: reactive oxygen species
NOX2: NADPH oxidase 2
Nrf2: NF-E2-related factor 2
MCAO: middle cerebral artery occlusion
GAP-43: growth associated protein-43
MAPK: mitogen-activated protein kinase
AchE: acetylcholinesterase
nuclear factor kappa-B
Aβ: amyloid β-protein
TLRs: toll-like receptors
JNK: c-Jun N-terminal kinase
MPTP: 1-methyl-4-phenyl-1,2,3,6-tetrahydropyridine
Wnt: wingless
RAGE: receptor for advanced glycation endproducts
AD: Alzheimer's disease
HD: Huntington's disease
PD: Parkinson's disease
OGD: oxygen glucose deprivation
VEGF: vascular endothelial growth factor
CSDS: chronic social defeat stress.
